# Metabolomics analysis of CEF cells infected with avian leukosis virus subgroup J based on UHPLC-QE-MS

**DOI:** 10.1016/j.psj.2024.103693

**Published:** 2024-03-28

**Authors:** Menglu Xu, Kun Qian, Hongxia Shao, Yongxiu Yao, Venugopal Nair, Jianqiang Ye, Aijian Qin

**Affiliations:** ⁎ Ministry of Education Key Lab for Avian Preventive Medicine, Yangzhou University, Yangzhou, Jiangsu, 225009, P.R. China; † Jiangsu Co-innovation Center for Prevention and Control of Important Animal Infectious Diseases and Zoonoses, Yangzhou, Jiangsu, 225009, P.R. China; ‡ The Pirbright Institute & UK-China Centre of Excellence on Avian Disease Research, Pirbright, Surrey, GU24 0NF, United Kingdom

**Keywords:** avian leukosis virus subgroup J, metabolite, lipid, CEF cell

## Abstract

Avian leukosis virus subgroup J (**ALV-J**) is a retrovirus that can cause immunosuppression and tumors in chicken. However, relative pathogenesis is still not clear. At present, metabolomics has shown great potential in the screening of tumor metabolic markers, prognostic evaluation, and drug target design. In this study, we utilize an untargeted metabolomics approach based on ultrahigh-performance liquid chromatography–quadrupole time-of-flight tandem mass spectrometry (**UHPLC-QTOF-MS**) to analyze the metabolic changes in chicken embryo fibroblast (**CEF**) cells infected by ALV-J. We found that ALV-J infection significantly altered a wealth of metabolites compared with control group. Additionally, most of the differentially expressed metabolites belonged to lipid metabolism, purine nucleotide metabolism and amino acid metabolism. Among them, the proportion of lipid metabolites account for the highest proportion (around 31%). Results suggest that these changes may be conductive to the formation of virion, thereby promoting the replication of ALV-J. These data provided metabolic evidence and potential biomarkers for the cellular metabolic changes induced by ALV-J, and provided important insight for further understanding the replication needs and pathogenesis of ALV-J.

## INTRODUCTION

Avian leukosis virus (**ALV**) is a retrovirus that can induce various malignant tumors such as myeloma, hemangioma, and connective tissue tumor, causing severe immune damage to the host (Rubin, 2011; Guo et al., 2014; Lipsick, 2021). ALV is divided into 11 subgroups based on the specificity of the glycoprotein gp85 on the viral envelope (Mo et al., 2022), the cross reaction patterns between virus and host factors (Cui et al., 2014). Seven subgroups including A, B, C, D, E, J, and K originate from chickens, with A, B, C, D, J, and K being exogenous viruses (Wang et al., 2012) and E being endogenous viruses (Su et al., 2018). Avian leukosis virus subgroup J (**ALV-J**) was identified in the 1988 and became a major concern in the poultry industry (Payne et al., 1991). Avian leukosis virus subgroup J strains such as the prototype strain HPRS103 in European and ADOL Hc1 in American usually induce myeloma in broiler chickens. However, ALV-J strains in China and Southeast Asia, have evolved into various strains with different pathological characteristics in broilers and layers (Wu et al., 2022). Compared with other subgroups, ALV-J has stronger pathogenicity and transmission ability, causing huge economic losses to industry (Ma et al., 2020; Xu et al., 2022). Although ALV-J eradication program have been successfully applied in China, ALV-J infection still spreads rapidly and persists long time. At present, little is known about the infection and pathogenic mechanisms of ALV-J. Therefore, searching for potential biomarkers provides a new perspective for the prevention of ALV-J.

In recent years, metabolomics technology has rapidly developed (Wishart, 2016) and widely applied to study the changes in virus metabolism in host cells, providing new insights for studying the pathogenic mechanism and treatments of viruses (Olund Villumsen et al., 2021; Xue et al., 2022). The viral infection causes significant changes in multiple metabolic pathways in host animals or cells, such as carbohydrate, fatty acid, and nucleotide metabolism, which are closely related to viral infection and replication processes (Wan et al., 2017). During viral infection, changes in cellular metabolism contribute to the survival of infected cells. For example, Hepatitis C virus infection can lead to disorders in glucose utilization and lipid biosynthesis (Blackham et al., 2010). After Zika virus infection in human cells, glucose consumption in the tricarboxylic acid cycle increases, selectively depleting triphosphate nucleotide levels, leading to an increase in AMP/ATP ratio, AMP activated protein kinase (**AMPK**) phosphorylation, and caspase-mediated cell death (Thaker et al., 2019). E4ORF1, the gene product of adenovirus, is necessary for adenovirus induced upregulation of glucose metabolism in host cells, and enhances glycolysis in primary lung epithelial cells by activating the oncogene (**MYC**), which is beneficial for adenovirus replication (Thai et al., 2014). After enterovirus infection in Vero cells, changes in glutathione metabolism, glycolysis, tricarboxylic acid cycle, and several amino acid libraries occurred in the infected cells. Glutamine lyases (**GLS/GDH**) and dihydroorotase (**CAD**) play important roles in meeting the metabolic needs of viral replication. Knocking down these enzymes can reduce cytopathic effects and the synthesis of viral genomic RNA (Cheng et al., 2020).

The metabolic alterations induced by different viruses are distinct. To the best of our knowledge, metabolomics studies have not yet been conducted in cells infected with ALV-J. In this study, we used untargeted ultrahigh-performance liquid chromatography–quadrupole time-of-flight tandem mass spectrometry (**UHPLC-QTOF-MS**) technology for metabolomics analysis of ALV-J infected cells. The results showed that a large number of metabolites and metabolic pathways changed significantly during ALV-J infection compared to uninfected cell control groups. This suggests that these changes may be in favor of viral replication, providing a basis for elucidating the pathogenic mechanism of ALV-J.

## MATERIALS AND METHODS

### Cell Culture and Virus

Chicken embryo fibroblast (**CEF**) cells were prepared from 9-day-old embryos as described previously (Zai et al., 2022). CEF cells were grown in Dulbecco's Modified Eagle's Medium (**DMEM**) (Thermo Fisher Scientific, Shanghai, China) supplemented with 5% fetal bovine serum (Thermo Fisher Scientific, Shanghai, China), 10% tryptone phosphate broth (Sigma, Shanghai, China), and 100 µg/mL of penicillin and streptomycin (Thermo Fisher Scientific, Shanghai, China) at 37°C in 5% CO_2_ atmosphere.

The ALV-J strain JSYC2106-1 (GenBank: OL799231.1) used in this experiment is kept in our laboratory.

### Virus Infection

The prepared CEF cells were seeded in a 6-well cell plate and incubated for 24 to 36 h at 37°C with 5% CO_2_. When the cell density reached approximately 80%, cells were infected with ALV-J at a multiplicity of infection (**MOI**) of 0.1 and incubated at 37°C for 2 h. After 2 h, the culture medium was replaced with fresh DMEM supplemented with 1% FBS. Cell samples were harvested at 0, 48, and 72 h post infection (**hpi**).

### Western Blot

Cell samples were harvested at 0, 48, and 72 hpi for immunoblotting analysis, which was performed as previously described (He et al., 2021). The target proteins were transferred to nitrocellulose membranes (Sigma, Shanghai, China) after SDS-PAG, the membrane was blocked with 5% skim milk in Tris-buffered saline-Tween (**TBST**) at room temperature for 2 h. Following the incubation with primary monoclonal antibody (mAb) against ALV-J P27 (Zhou et al., 2019) and anti-α-tubulin mAb (Sigma, Shanghai, China), and the secondary antibody horseradish peroxidase (**HRP**)-conjugated goat anti-mouse IgG (Jackson ImmunoResearch Laboratories, West Grove, PA), the protein bands were visualized using a chemiluminescence detection reagents (NCM Biotech, Newport, RI).

### Immunofluorescence Assay

Cell samples were harvested at 0, 48, and 72 hpi for immunofluorescence assay (**IFA**) which was performed as previously described (He et al., 2021). The fixed cells were incubated with anti-P27 mAb prior incubating with FITC-labelled goat anti-mouse antibodies IgG (Jackson ImmunoResearch Laboratories) at 37°C for 45 min. Images were captured with an OLYMPUS fluorescence microscope.

### Sample Preparation and Metabolite Extraction

CEF cells were infected with ALV-J (JSYC2106-1 strain) at MOI of 0.1. Cell samples were collected at 0, 48, and 72 h. Samples at 0 h time points were regarded as the control group. Cells were counted to ensure that samples in each group were no less than 1 × 10^7^ cells with 4 replicates per group. Cell samples were centrifuged at 1,000 × *g* for 5 min at 4°C. The cell pellets were quickly frozen in liquid nitrogen for 30 s, and then stored at -80°C. For metabolite extraction, the frozen samples were thawed slowly at 4°C followed by adding 1,000 μL of methanol/acetonitrile/H2O (2:2:1, v/v/v) to homogenized solution. Following centrifugation of the mixture for 15 min (14,000 *g*, 4°C), the supernatant was dried in a vacuum centrifuge. For LC-MS analysis, the samples were re-dissolved in 100 μL acetonitrile/water (1:1, v/v) solvent.

### UHPLC-QE-MS Analysis and Statistical Analysis

The UHPLC-QE-MS analysis and data analysis were performed as described previously (Ma and Niu, 2021) using an UHPLC (1290 Infinity LC, Agilent Technologies, Santa Clara, CA) coupled to a quadrupole time-of-flight (AB Sciex TripleTOF 6600). After normalization to total peak intensity, the processed data were analyzed by R package (ropls), where it was subjected to multivariate data analysis, including Pareto-scaled principal component analysis (**PCA**) and orthogonal partial least-squares discriminant analysis (**OPLS-DA**). The variable importance in the projection (**VIP**) value of each variable OPLS-DA model was calculated to indicate its contribution to the classification. Metabolites with the VIP value >1 was further applied to Student's *t*-test at univariate level to measure the significance of each metabolite, the *p* values less than 0.05 were considered as statistically significant.

### Total RNA Extraction and Quantitative Real-Time PCR Analysis

Cell samples were collected after ALV-J infection of CEF cells with MOI of 0.1. Meanwhile, uninfected cells were used as control. Total RNAs from cell samples were extracted using FastPure Cell/Tissue Total RNA Isolation Kit (Vazyme, Nanjing, China). Reverse transcription was performed using HiScript III first strand cDNA synthesis kit (Vazyme, Nanjing, China) for cDNA preparation. The SYBR qPCR Master Mix (Vazyme, Nanjing, China) were used for quantitative real-time reverse transcriptase–PCR. All experiments were performed in triplicate. The Gene expression levels were quantified relative to GAPDH with the comparative threshold cycle 2^−ΔΔCT^ method. The primer sequence is shown in Table 1.

**Table 1:** Sequences of primers used in this study.

Primer name	Sequence (5′-3′)
GAPDH-F	AGGGTGGTGCTAAGCGTGTTA
GAPDH-R	TCTCATGGTTGACACCCATCA
NAAA-F	TGAGTGCTGCATTTCCAGAGA
NAAA-R	TTTCTCCTATTCAGTCAGCATGT
CHKA-F	CTCATCAGCCCCATCAGAGG
CHKA-R	CATGGCTTCTGCCCCCATCT
ASAH1-F	CTCCTTCAGGACCAACGTTCA
ASAH1-R	GGCACGTATCAACTCATCCCA
SGPP1-F	GGTGAAGCTGGAGGTCTTCTACA
SGPP1-R	AACACAAGGGGATACTGCCAGC
SMPD1-F	CCGCATCGTGAACAGGTTTG
SMPD1-R	GAGTCAGCGTCTCCTCATCG
PGS1-F	AGACTGGGGATTGAAGAACCC
PGS1-R	CGCTTTGCTCTGCAGTACTTT
DEGS2-F	CAGGAAGGAGATCCTGGCAAAA
DEGS2-R	AACTGCATGAAAACCATTCCAGA

## RESULTS

### Replication of ALV-J in CEF Cells

**Figure 1:**
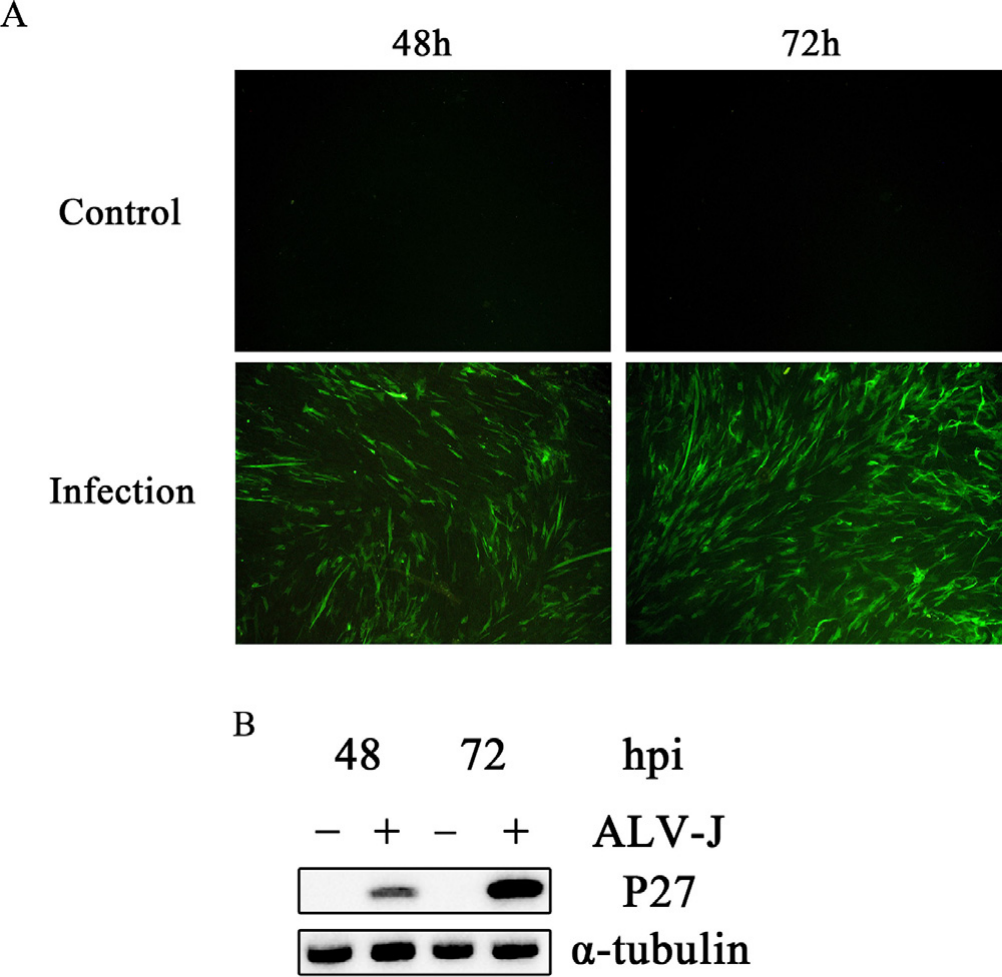
CEF cells were infected with ALV-J at different time points. (A) CEF cells were infected with ALV-J (MOI = 0.1) for 0, 48, 72 h. Cell samples were collected for IFA to detect P27 expression. (B) Expression of viral P27 protein was detected by Western blotting. α-tubulin expression was used as control.

The cell samples collected at 48 h and 72 h after infection with ALV-J (JSYC2106-1) strain were processed for ALV-J infection status by IFA and Western blotting with anti-P27 mAb. The results showed that the intensity of fluorescence in ALV-J infected CEF cells were progressively stronger and reached a high level at 72 hpi. (Figure 1A). With α-tubulin as a loading control, Western blot results showed that the expression of viral protein P27 also increased with time (Figure 1B). These results confirmed that ALV-J could replicate effectively in CEF cells within 72 h of infection.

### Multivariate Analysis

**Figure 2:**
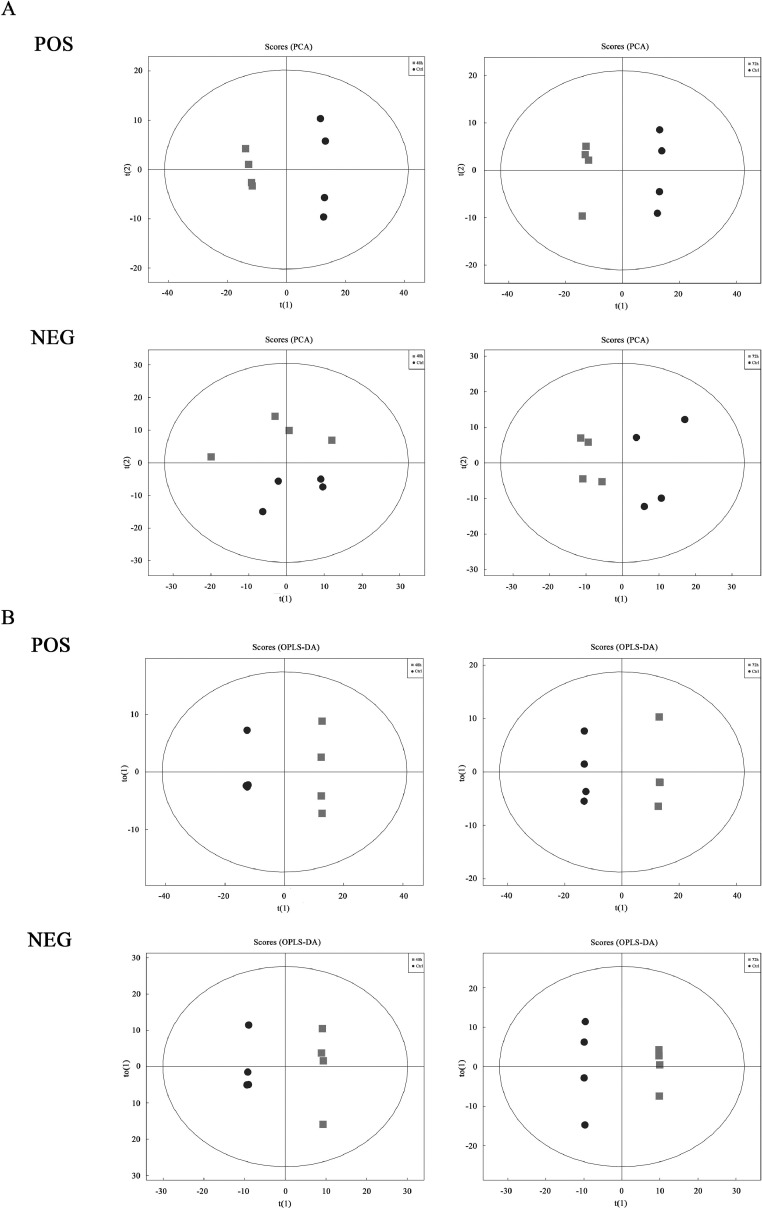
Score plots of PCA and OPLS-DA of CEF cells infected with ALV-J. Score scatter plot of the PCA (A) and OPLS-DA (B) model for the infection groups at different time points vs. control group. Electrospray ionization served as the source of UHPLC-QE-MS, including positive and negative ion modes (POS and NEG).

Electrospray ionization is the source of UHPLC-QTOF-MS and contains positive (**POS**) and negative (**NEG**) ion modes. PCA was performed to identify the overall distribution trend and the degree of difference between the samples. PCA scatter plot result showed that all samples were within the 95% confidence interval, with differences in distribution between groups (Figure 2A). OPLS-DA could directly differentiate inter-group variation from x-axis, while intra-group variation was reflected in y-axis (Figure 2B). The evaluation parameter of the OPLS-DA model (Table 2). The parameters of the OPLS-DA evaluation model include R^2^X, R^2^Y, and Q^2^, where R^2^X and R^2^Y represent the explanatory power of the constructed model to the X and Y matrices, Q^2^ represents the predictive ability of the model, and Q^2^>0.5 indicates the reliability of the mode. These results indicate that different groups are clearly distinguishable, and these models are efficient and reliable.

**Table 2:** Parameters of OPLS-DA model between the groups.

		POS			NEG		
Title	Type	R^2^X(cum)	R^2^Y(cum)	Q^2^(cum)	R^2^X(cum)	R^2^Y(cum)	Q^2^(cum)
48hpi	OPLS-DA	0.628	1	0.978	0.349	1	0.578
72hpi	OPLS-DA	0.663	1	0.98	0.353	1	0.669

### Differential Metabolites Analysis

**Figure 3:**
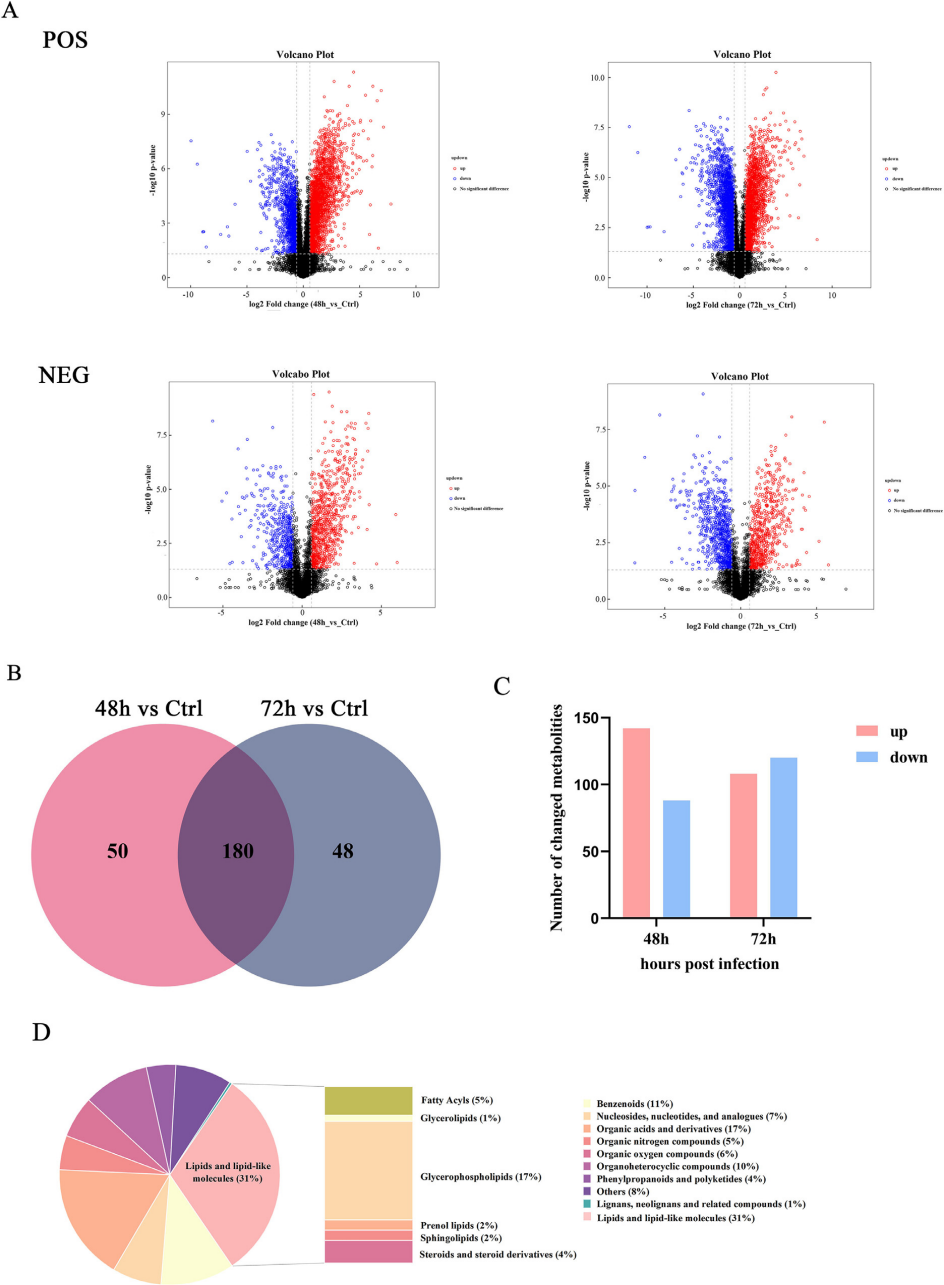

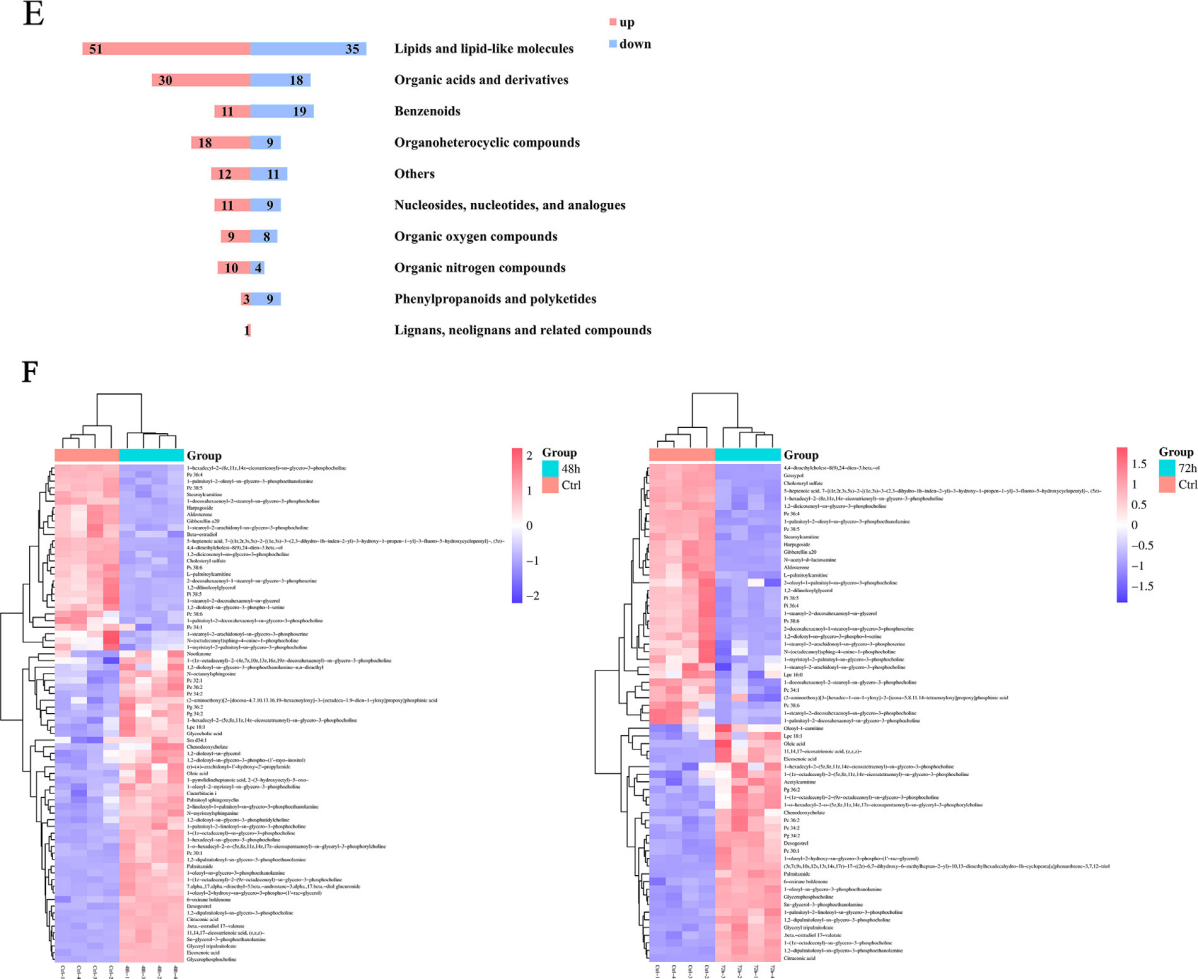
Analysis of differentially expressed metabolites in ALV-J infected CEF cells. (A) Volcano plots for the control-infected and ALV-J-infected cells at 48 h and 72 h. Red, upregulation; blue, downregulation; gray, unchanged. (B) Venn plot of ALV-J infection group (48h, 72h) and control group. (C) Numbers of differentially expressed metabolites upregulated (red) and downregulated (blue) in infected groups. (D, E) Pie charts and histograms show the proportion of different categories of metabolites differentially expressed in ALV-J infected CEF cells. (F) Heat map of hierarchical clustering analysis of differential metabolites. Each column represents one sample, and each row represents one differential metabolite. Red, upregulation; blue, downregulation.

The Volcano Plot shows 2 important indicators in one graph (fold changes of > 1.5 or < 0.67, *P* < 0.05), with clear identifiable differentially expressed genes between the 2 samples. All metabolites detected in positive and negative ion mode (containing unidentified metabolites) were analyzed (Figure 3A). Each circled dot in the volcanic map represented a metabolite. The VIP value >1 of the OPLS-DA model and the *P* value < 0.05 were used as the criteria for significant differential metabolite screening. The numbers of differential metabolites in CEF cells infected with ALV-J were 230 and 228 at 48 hpi and 72 hpi respectively (Figure S1), and the shared differential metabolites were 180 (Figure 3B). Among them, the most of metabolite fraction was associated with lipids and lipid-like molecules (Figure S2). A total of 142 and 108 metabolites were up-regulated, 88 and 120 metabolites were down-regulated in ALV-J infected cells compared to the control group at 48 hpi and 72 hpi, respectively (Figure 3C). In addition, classification analysis of these differential metabolites showed that Lipids and lipid-like molecules accounted for the highest proportion of differential metabolites in ALV-J infected cells, accounting for approximately 31%. Among them, glycerophospholipids showed the most significant changes, followed by Fatty Acyls, Steroids and steroid derivatives (Figures 3D and 3E). In order to investigate the key lipid metabolites related to the ALV-J replication process, we screened 2 groups of lipid differential metabolites (48 hpi vs. control and 72 hpi vs. control). Compared with control, 75 and 65 lipid related metabolites were detected at 48hpi and 72hpi respectively, as presented in the heatmap of hierarchical clustering analysis (Figure 3F). As expected, metabolites changed with viral infection. These results indicated that lipid metabolism in host cells may play an important role in ALV-J replication.

### Metabolic Pathway Analysis

**Figure 4:**
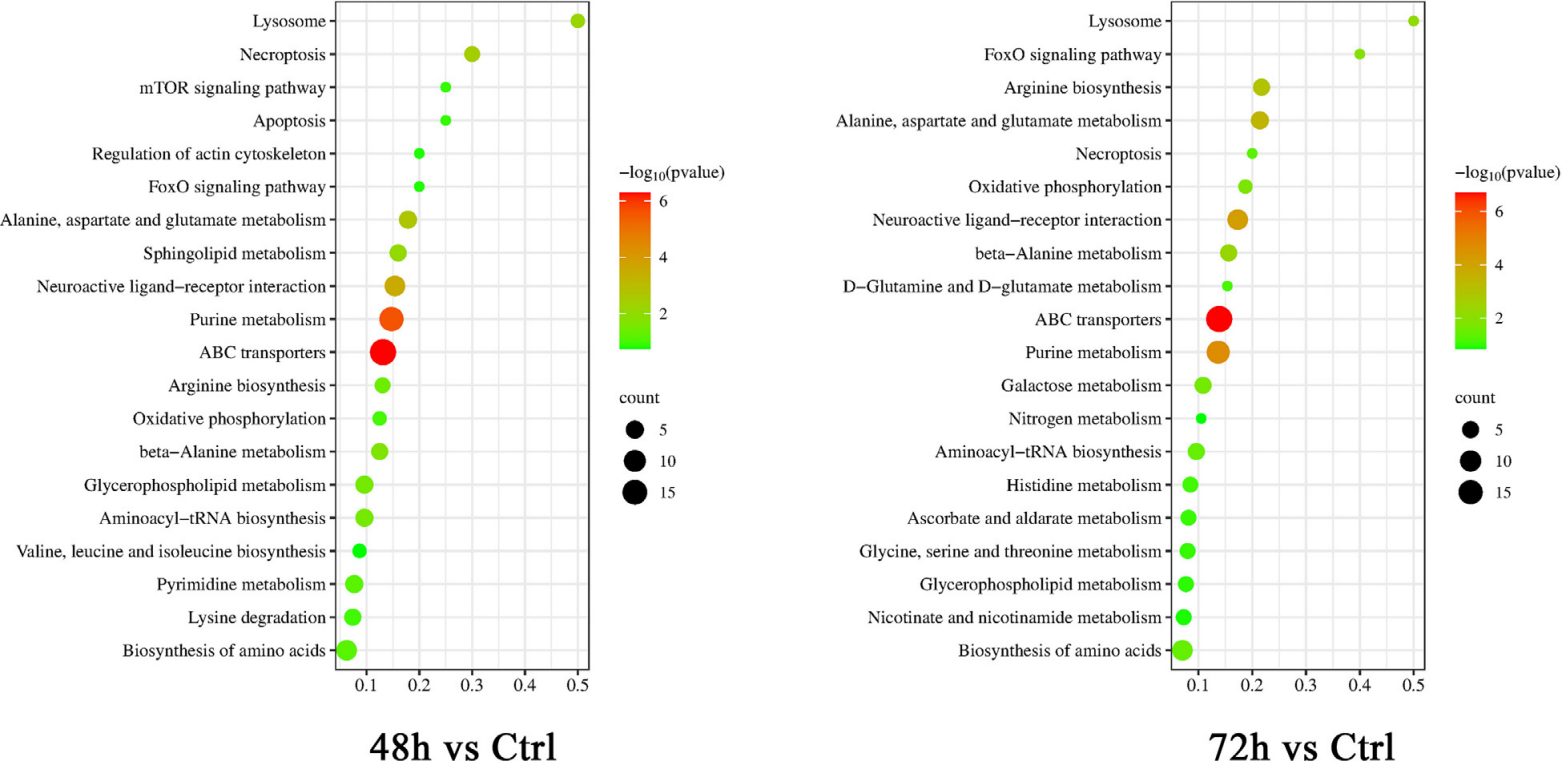
Bubble plot analysis of metabolic pathways in ALV-J-infected CEF cells. Each bubble in the bubble chart represents a metabolic pathway (the 20 most significant ones were displayed according to the *P*-value). The larger the bubble, the more metabolites it contains. The x-axis represents the path influence value in topology analysis, and its size is positively correlated with the influence factor. The y-axis represents the *P*-value of metabolic pathways in enrichment analysis; The darker the color and the smaller the *P*-value indicate a more significant degree of enrichment.

KEGG metabolome database was used to annotate these differential metabolites and find metabolic pathways with high correlation. The top 20 metabolic pathways with the highest significance were selected according to the *p*-value, and the results are shown in Figure 4. Compared with the control group, the differential metabolites in the infection group were mainly enriched in ABC transporters, Purine metabolism, Neuroactive ligand-receptor interaction, Alanine, aspartate and glutamate metabolism, necroptosis, lysosome, sphingolipid metabolism, glycerophospholipid metabolism, and arginine biosynthesis.

### Validation of the Lipid Metabolism

**Figure 5:**
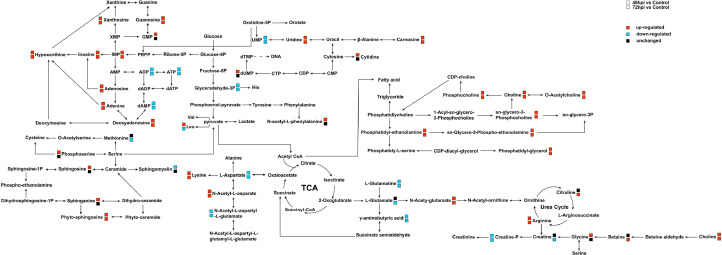
Schematic overview of altered metabolic pathways in ALV-J infected CEF cells. The metabolites were shown in different colors according to their changes. Red: upregulated; Blue: downregulated; Black: unchanged.

Our results present visualized profiles of the metabolite changes in CEF cells infected with ALV-J. Some metabolites with significant changes were further screened to establish connections among the related metabolic pathways (Figure 5). These metabolic pathways include TCA cycle, lipid metabolism, amino acid metabolism, and nucleotide metabolism. The metabolic pathways of glycerophospholipids and sphingolipids were reprogrammed at 48 and 72 hpi in ALV-J-infected cells, and the levels of phosphocholine, phospholipid-ethanolamine, choline, sphingosine, and sphinganine increased with the ALV-J replication process, which indicated that they may be required in the virus replication cycle. There were also significant changes in nucleotide metabolism during ALV-J infection. Compared to the control group, the levels of hypoxanthine nucleotide (**IMP**), inosine, hypoxanthine, adenosine and deoxyadenosine were significantly upregulated, which is consistent with virus replication. In addition, some amino acids were also changed after ALV-J infection. These results indicated that ALV-J infection leads to metabolic reprogramming in CEF cells, which facilitates virus replication.

### Validation of Metabolomic Data by qPCR

Considering the important role that lipid metabolism may play in ALV-J infection, cell samples were collected after ALV-J infection of CEF cells with MOI of 0.1 at 48 and 72 hpi for further validation of metabolomics data. The gene levels of enzymes related to glycerol phospholipid metabolism and sphingolipid metabolism, including N-acyl-sphingosine amidohydrolase 1 (**ASAH1**), N-acyl-sphingosine acid amidase (**NAAA**), choline kinase alpha (**CHKA**), phosphotidyl-glycerophosphate synthase 1 (**PGS1**), sphingosine 1-phosphate phosphatase 1 (**SGPP1**), delta 4-characterize sphingolipid 2 (**DEGS2**), sphingosine 1-phosphate gomyelin-photoshodiesterase 1 (**SMPD1**) were analyzed by qPCR (Figure 5). The result showed that during ALV-J infection, the levels of ASAH1, NAAA, CHKA, PGS1, SGPP1, DEGS2, and SMPD1 genes were significantly increased with infection time. The above results indicated that when ALV-J infects cells, some lipid metabolism pathways within the host cell are promoted, thereby promoting virus replication.

## DISCUSSION

In recent years, metabolomics methods have been applied to the study of various viral infections. These methods involve the determination and comparison of a large number of metabolites in biological samples using analytical substance techniques, combined with high-throughput analytical chemistry and multivariate data analysis, to study the metabolic effects of viruses during in vitro replication (Mahmud and Garrett, 2020). Viral induced metabolism can provide the nucleotides required for rapid replication of the viral genome and the amino acids required for rapid assembly of viral particles. Envelope viruses require more lipids to provide additional membrane materials to encapsulate virus particles. It is necessary to determine how viruses alter cellular metabolism and how these metabolisms change during the virus lifecycle, which will help deepen our understanding of the replication needs of viruses and may provide cellular targets for virus inhibition (Sreekumar et al., 2009). Therefore, in order to understand the metabolic changes of ALV-J infected cells, this study analyzed the metabolomics characteristics of CEF cells infected with ALV-J based on UHPLC-QTOF-MS. These results provide new insights into the interaction between ALV-J and host cells, which helps to determine the infection mechanism of ALV-J.

In this study, we analyzed data on the changes in metabolites and related pathways in CEF cells after ALV-J infection. The results of OPLS-DA and hierarchical clustering analysis showed that compared with the control group, the infected cells exhibited significantly different metabolic characteristics, leading to metabolic reprogramming. Overall, significant changes have occurred in lipid metabolism and nucleotide metabolism, and these changes in metabolites and pathways reflect the cell response to ALV-J infection, which helps to understand how ALV-J regulates host metabolism to promote self-replication.

**Figure 6:**
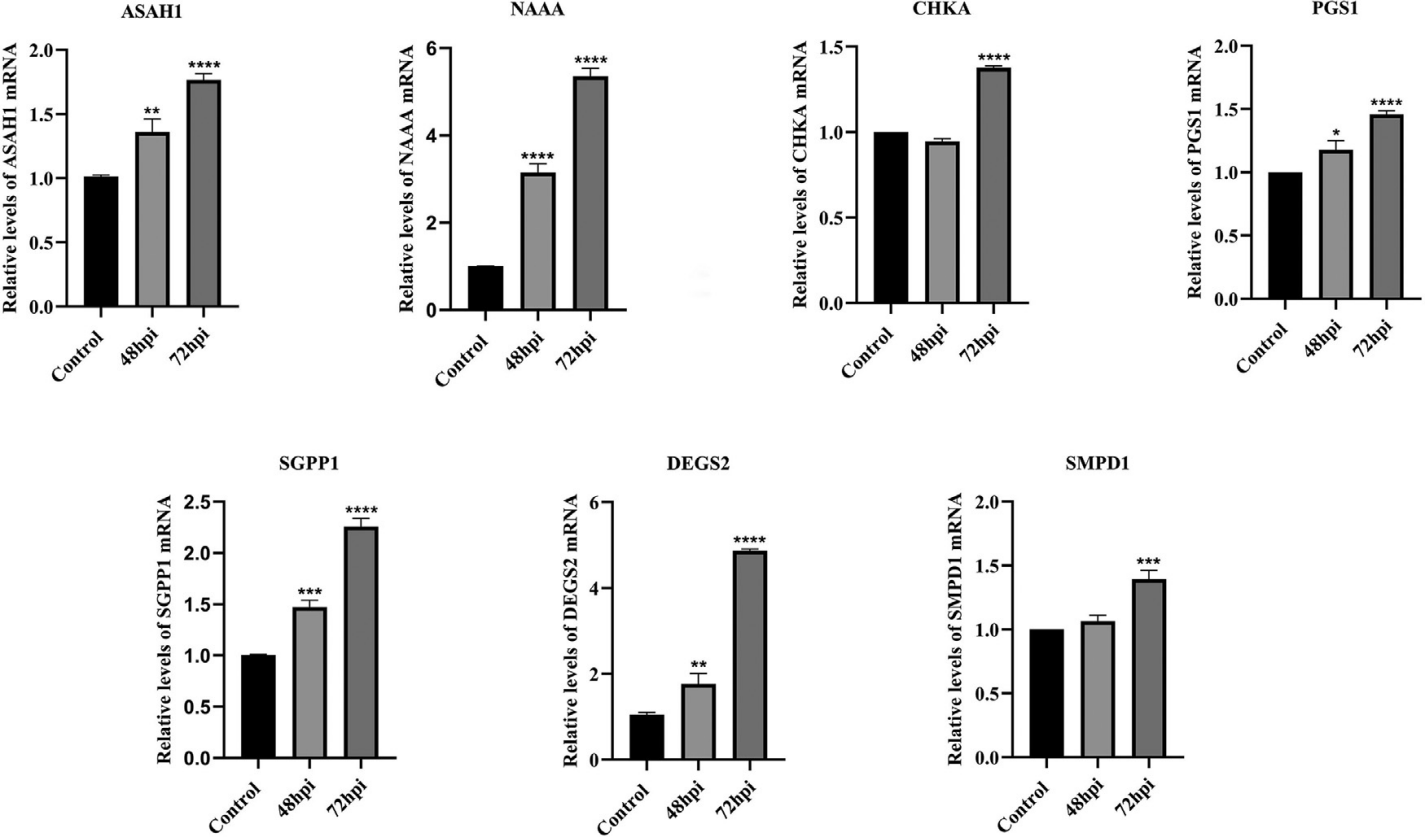
The mRNA levels of lipid-metabolism-related enzymes postinfection of ALV-J. CEF cells were infected with ALV-J at a MOI of 0.1 and harvested at 48 and 72 hpi postinfection, while noninfected cells were used as control.

Lipids play a very important role in the life cycle of viruses, as they can affect the host energy state, endocytosis, cell signaling, and virus assembly and budding (Getts et al., 2013; Moller-Tank and Maury, 2014). Lipids are the first barrier to infection, and similar to other metabolic pathways, viruses have evolved to utilize the host lipids for successful adhesion and entry (Schoggins and Randall, 2013; Roller and Johnson, 2021). Lipids mainly include 3 major categories: phospholipids, sphingolipids, and cholesterol (Harayama and Riezman, 2018). Studies have shown that herpesvirus infects host cells and disrupts their lipid metabolism, hijacking the host cell membrane system to complete virus replication (Gonzalez-Del Pino and Heldwein, 2022). Metabolomics analysis of cytomegalovirus (**CMV**) infection indicates an increase in the flow of metabolites entering the fatty acid biosynthesis pathway (Sanchez et al., 2017). Further research has found that inhibiting fatty acid biosynthesis can inhibit CMV infection (Yu et al., 2011). Hepatitis C virus (**HCV**) infection significantly alters intracellular phospholipid and sphingomyelin metabolism, thereby facilitating virus replication, assembly, and secretion (Diamond et al., 2010). Herpes simplex virus (**HSV-1**) can stimulate phospholipid synthesis in host cells and provide a membrane to form transport vacuole (Sutter et al., 2012). In our study, ALV-J, as a type of envelope virus, also requires a large amount of lipids to participate in the formation and release of virions. The increased availability of lipids during ALV-J infection could play a pivotal role in the rapid replication of the virus, by providing the needs of viral particle assembly. During ALV-J infection, the most metabolites fraction was associated with lipids and lipid-like molecules (Figure 3D). In addition, glycerol phospholipid metabolism and sphingolipids metabolism are significantly upregulated (Figure 3F and Figure 5). Similarly, our q-PCR results confirmed the upregulation of mRNA levels of lipid metabolism related enzymes during ALV-J infection (Figure 6). Among them, the mRNA level of ASAH1 and CHKA were significantly increased in CEF cells infected with ALV-J. ASAH1 belongs to acid ceramidase, which is a key enzyme in sphingolipid metabolism. It hydrolyzes ceramide to generate sphingosine. It has been reported that Ceranib-2, a specific acid ceramidase inhibitor, reduces SARS-CoV-2 replication (Geiger et al., 2022) and impair measles virus (**MV**) replication (Grafen et al., 2019). CHKA is an important enzyme for the biosynthesis of phosphocholine from choline. Previous studies have shown that hepatitis B virus (**HBV**) infection of host cells upregulates phosphocholine biosynthesis by activating CHKA. However, silencing the expression of CHKA can inhibit HBV replication, indicating CHKA could be potential target to HBV (Li et al., 2015). They showed that glycerolphospholipid metabolism and sphingolipids metabolism are essential for virus replication. In summary, during ALV-J infection, several core lipid metabolism pathways may be hijacked to provide necessary biosynthetic precursors and free energy, thereby maximizing the replication and assembly of the virus.

Purine metabolism and pyrimidine metabolism are the basic steps for nucleotide synthesis. In cancer and viral infections, host cells support their own proliferation and effector function by increasing nucleotide synthesis (Ariav et al., 2021). Human cytomegalovirus (**HCMV**) induces changes in the host metabolic network, and HCMV infection induces de novo biosynthesis flux of pyrimidine, thereby providing protein glycosylation and producing glycosyl subunits required for high-titer infectious offspring (DeVito et al., 2014). By activating MAPK dependent signaling pathway, KRAS leads to MYC up-regulation and transcription of nonoxidative pentose phosphate pathway (**PPP**) gene RPIA, thus promoting nucleotide biosynthesis and accelerating the development of pancreatic cancer (Santana-Codina et al., 2018). After ALV-J infection, there were significant changes in nucleotide metabolism, indicating an unknown role of ALV-J in replication (Figure 6), which may provide potential opportunities for the development of antiviral therapies.

There is currently limited research on host metabolism during ALV-J infection. Chen (2021) utilized liquid chromatography-mass spectrometry (**LC-MS**) analysis technology to explore the metabolic network of chicken plasma samples infected with ALV-J (Chen et al., 2021). They identified the top 15 metabolites screened as potential biomarkers for therapeutic targets of ALV-J induced diseases. Among them, 2-methylthiobenzothiazole, irinotecan, methadone, 3-o-ethyl-L-ascorbic acid, and o-acetylneuraminic acid showed a significantly increased levels and Methyl bromide, pyraclonil, hexaffumuron, lythidathion, 3-phosphoglycerol-glutathione, bis-4-nitrophenyl phosphate, 4-ketocyclophosphamide, oxidized photinus luciferin, phenyl sulfate, aryl sulfate showed a clearly decreased levels in ALV-J infected chickens compared to those in uninfected controls. However, these metabolites are not within the category of phospholipids. Due to the differences in the types of metabolites screened and samples (chicken plasma vs. CEF cells), our results differ from that report.

In conclusion, the metabolic changes of ALV-J (JSYC2106-1 strain) infected CEF cells were analyzed using UHPLC-QE-MS. There were significant differences in lipid metabolism and nucleotide metabolism between the infection group and the control group. Virus infection alters the metabolism of host cells, providing energy and material for virus replication. At present, inhibitors targeting lipid metabolism and nucleotide metabolism have been used to treat certain viral infections. Therefore, searching for metabolic targets for antiviral therapy may be a promising strategy.

## Appendix Supplementary materials

Image, application 1
