# Supplementary material for: Metabolomics analysis of CEF cells infected with avian leukosis virus subgroup J based on UHPLC-QE-MS
Source: Poult Sci. 2024 Mar 28;103(6):103693. doi: 10.1016/j.psj.2024.103693 (PMC11017069; doi:10.1016/j.psj.2024.103693)
Supplement: Supplementary file 1 [file mmc1.zip › mmc1/Supplementary.docx]

Figure S1. Pie graph show the distribution of metabolite superclasses. The metabolomics data included annotations for 230 differential metabolites (48 hpi) and 228 differential metabolites (72 hpi).

Figure S2. Pie chart show the distribution of the 180 metabolite superclasses in common, each section is colored to represent a certain class of metabolites.
