# Supplementary figures and images for: Metabolomics analysis of CEF cells infected with avian leukosis virus subgroup J based on UHPLC-QE-MS
Source: Poult Sci. 2024 Mar 28;103(6):103693. doi: 10.1016/j.psj.2024.103693 (PMC11017069; doi:10.1016/j.psj.2024.103693)

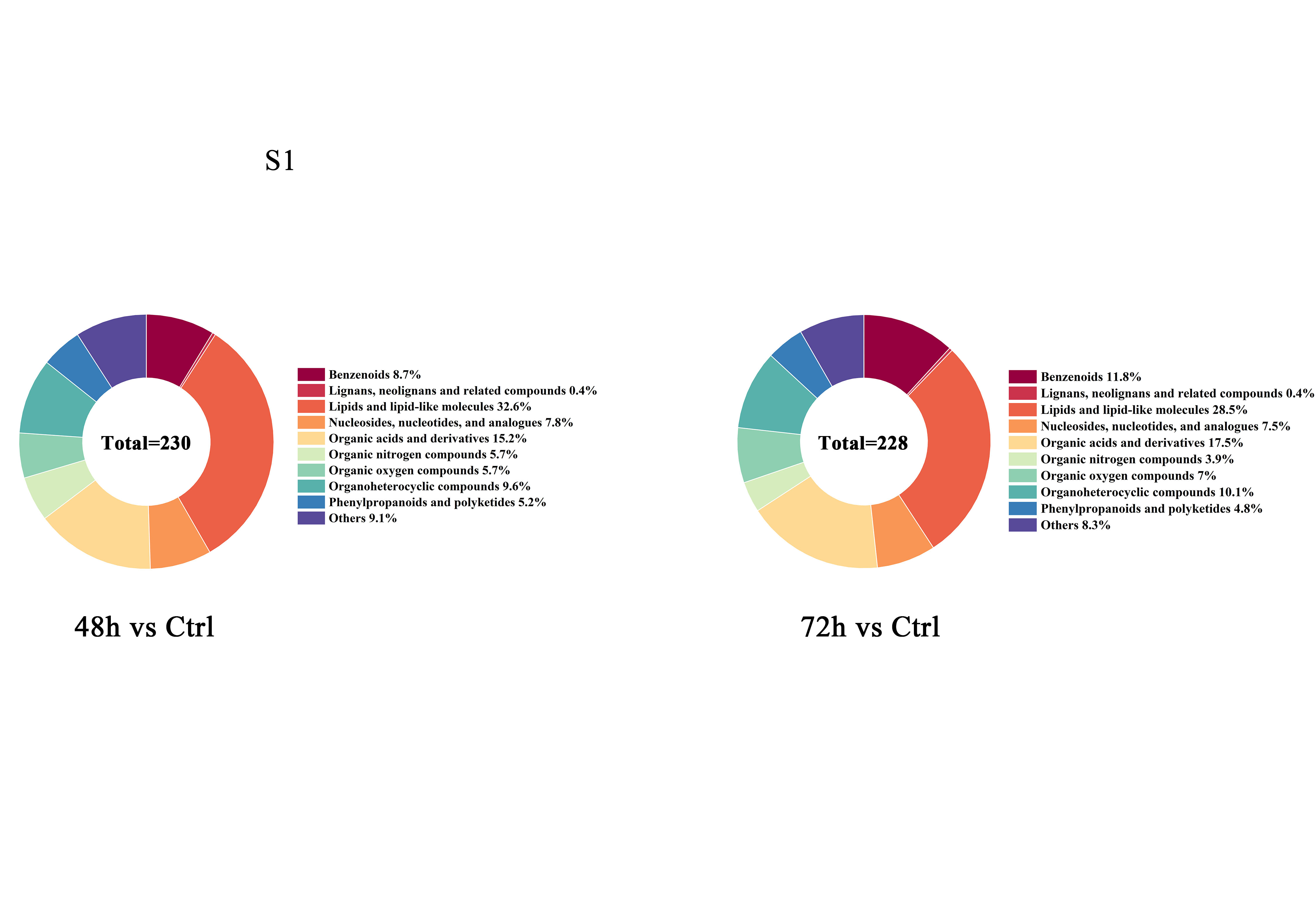

Supplement: Supplementary file 1 [file mmc1.zip › mmc1/Figure S1.jpg]

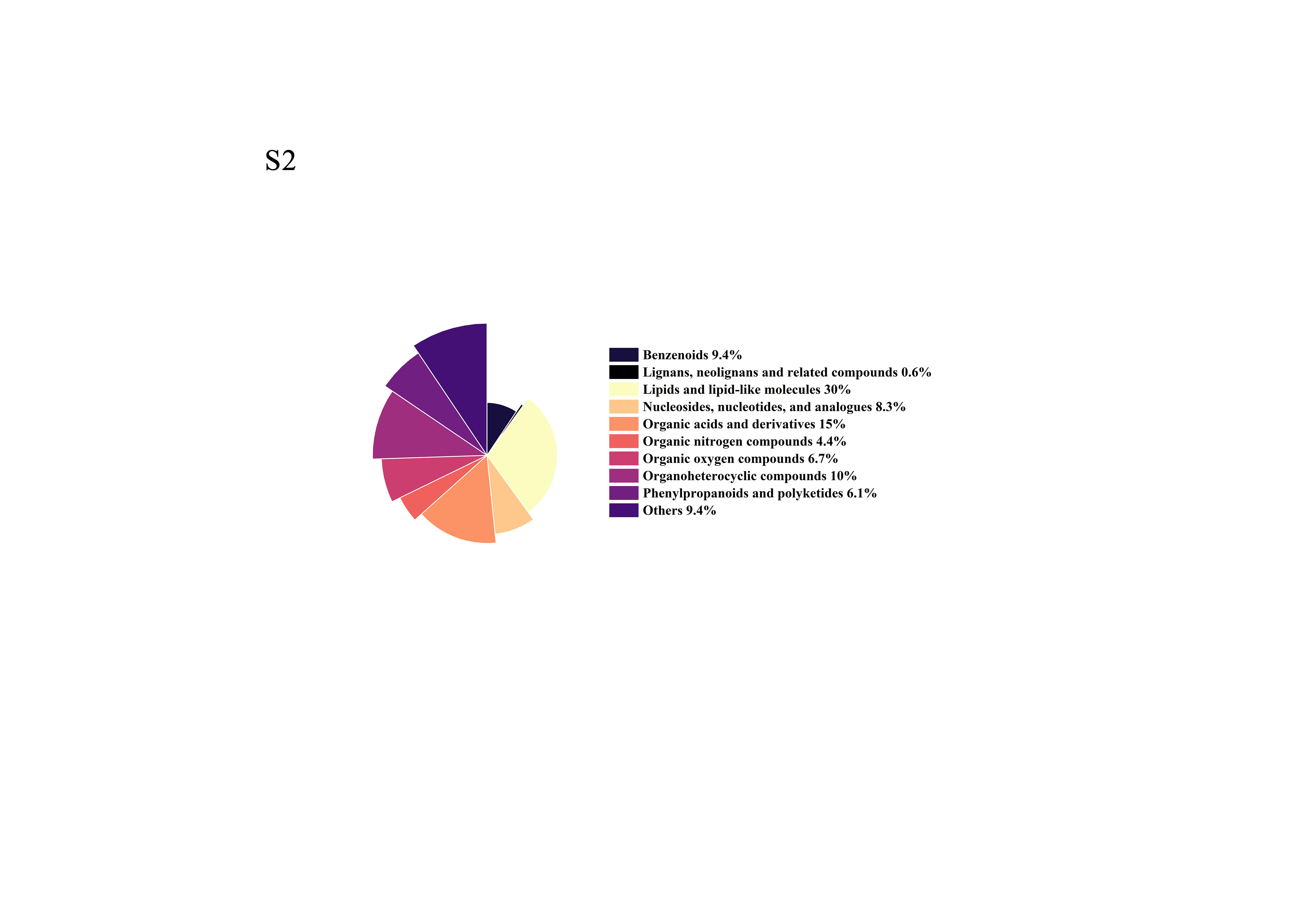

Supplement: Supplementary file 1 [file mmc1.zip › mmc1/Figure S2.jpg]
